# Supplementary material for: Modifiable Risk Factor Possession Patterns of Dementia in Elderly with MCI: A 4-Year Repeated Measures Study
Source: J Clin Med. 2020 Apr 10;9(4):1076. doi: 10.3390/jcm9041076 (PMC7230268; doi:10.3390/jcm9041076)
Supplement: Supplementary file 1 [file jcm-09-01076-s001.docx]

# Supplementary Table 1. Baseline characteristics in participants who remained versus left the follow up (drop-out).

|  | Participants who remained the follow-up (n = 461) | Participants who left the follow-up (n =328) | P value |
| --- | --- | --- | --- |
| Age (years)* | 70.9 (4.5) | 73.5 (6.1) | <0.01 |
| Sex (% male) | 48.4 | 44.2 | >0.05 |
| Education (years)* | 11.6 (2.5) | 10.8 (2.4) | <0.01 |
| Heart disease (% yes) | 15.8 | 18.9 | >0.05 |
| Pulmonary disease (% yes) | 8.2 | 10.7 | >0.05 |
| Walking speed (m/s)* | 1.3 (0.2) | 1.2 (0.2) | <0.01 |
| Mini-mental state examination (points)* | 26.6 (1.9) | 26.4 (1.8) | >0.05 |
| Geriatric depression scale (points)* | 2.7 (2.4) | 3.6 (2.8) | <0.01 |
| **Category of MCI (%)** |  |  | >0.05 |
| amnestic MCI single domain | 14.5 | 13.7 |  |
| non-amnestic MCI single domain | 64.4 | 58.5 |  |
| amnestic MCI multiple domain | 5.9 | 9.8 |  |
| non-amnestic MCI multiple domain | 15.2 | 18.0 |  |
| **Modifiable risk factors (% yes)** |  |  |  |
| Less education (10 years ≥) | 30.6 | 41.8 | <0.01 |
| Hearing loss | 2.4 | 5.9 | <0.05 |
| Hypertension | 48.2 | 46.6 | >0.05 |
| Obesity (BMI 25≤) | 29.0 | 26.5 | >0.05 |
| Smoking | 41.0 | 38.7 | >0.05 |
| Depression (GDS 5≤) | 18.7 | 30.0 | <0.01 |
| Physical inactivity | 29.5 | 32.3 | >0.05 |
| Social isolation | 30.2 | 45.6 | <0.01 |
| Diabetes | 15.0 | 14.6 | >0.05 |

* Mean (Standard Deviation)
